# A randomised controlled trial of the effect of providing online risk information and lifestyle advice for the most common preventable cancers: study protocol

**DOI:** 10.1186/s12889-018-5712-2

**Published:** 2018-06-26

**Authors:** Juliet A. Usher-Smith, Golnessa Masson, Katie Mills, Stephen J. Sharp, Stephen Sutton, William M. P. Klein, Simon J. Griffin

**Affiliations:** 1 The Primary Care Unit, Institute of Public Health, University of Cambridge School of Clinical Medicine, Box 113 Cambridge Biomedical Campus, Cambridge, CB2 0SR UK; 2 MRC Epidemiology Unit, Institute of Metabolic Science, University of Cambridge, Cambridge, CB2 0QQ UK; 3 Behavioural Science Group, The Primary Care Unit, Institute of Public Health, University of Cambridge School of Clinical Medicine, Box 113 Cambridge Biomedical Campus, Cambridge, CB2 0SR UK; 4 Behavioral Research Program, National Cancer Institute, Rockville, MD USA

**Keywords:** Risk, Cancer, Risk perception, Behaviour, Communication, Protocol, Randomised controlled trial

## Abstract

**Background:**

Cancer is a leading cause of mortality and morbidity worldwide. Prevention is recognised by many, including the World Health Organization, to offer the most cost-effective long-term strategy for the control of cancer. One approach that focuses on individuals is the provision of personalised risk information. However, whether such information motivates behaviour change and whether the effect is different with varying formats of risk presentation is unclear. We aim to assess the short-term effect of providing information about personalised risk of cancer in three different formats alongside lifestyle advice on health-related behaviours, risk perception and risk conviction.

**Methods:**

In a parallel group, randomised controlled trial 1000 participants will be recruited through the online platform Prolific. Participants will be allocated to either a control group receiving cancer-specific lifestyle advice alone or one of three intervention groups receiving the same lifestyle advice alongside their estimated 10-year risk of developing one of the five most common preventable cancers, calculated from self-reported modifiable behavioural risk factors, in one of three different formats (bar chart, pictograph or qualitative scale). The primary outcome is change from baseline in computed risk relative to an individual with a recommended lifestyle at three months. Secondary outcomes include: perceived risk of cancer; anxiety; cancer-related worry; intention to change behaviour; and awareness of cancer risk factors.

**Discussion:**

This study will provide evidence on the short-term effect of providing online information about personalised risk of cancer alongside lifestyle advice on risk perception and health-related behaviours and inform the development of interventions.

**Trial registration:**

ISRCTN17450583. Registered 30 January 2018.

**Electronic supplementary material:**

The online version of this article (10.1186/s12889-018-5712-2) contains supplementary material, which is available to authorized users.

## Background

Cancer is now a leading cause of mortality and morbidity worldwide [1]. Prevention is recognised by many, including the World Health Organisation [2], to offer the most cost-effective long-term strategy for the control of cancer. It is estimated that approximately 40% of cases of cancer are attributable to lifestyle factors including smoking, diet, alcohol consumption, physical activity and weight [3]. Prevention strategies targeting these behaviours are likely to require a combination of collective approaches aimed at shifting the population distribution and approaches that focus on individuals.

One element of approaches that focus on individuals is the provision of risk information. Many behaviour change theories suggest that to engage in risk-reducing behaviour, individuals must believe that they are at risk [4, 5]. Although evidence for behaviour change following provision of risk information in general is limited [6–8], in a recent systematic review of randomised controlled trials of the effect of interventions incorporating personalised non-genetic cancer risk information on intentions and behaviour [9], we found only one study that reported the effect on smoking status [10] and none assessing the impact on diet, physical activity and alcohol consumption. The impact of cancer risk information on lifestyle behaviour is, therefore, not known.

Additionally, while it has been demonstrated that providing individuals with information about their risk of cancer can improve accuracy of risk perception [11–13], studies have tended to focus on risk perception as a deliberative, reason-based concept. More recent work has highlighted the distinction between deliberative, affective and experiential risk perceptions [14, 15]. Instead of being driven by probability judgments or deliberation, affective risk perceptions reflect an emotional response to a threat, such as fear or worry, and experiential risk perceptions involve “gut-level reactions” based on learned associations [15]. A tripartite model (TRIRISK) including these three components has recently been developed, and confirmatory factor analyses have shown it to provide a better fit to the data than either dual-factor or single-factor models [15]. Distinguishing in this way between deliberative, affective, and experiential risk perceptions therefore has the potential to provide greater insights into associations between risk perception and behaviour change. No studies have yet been published in which these three components have been measured following the provision of risk information.

Risk conviction, the certainty and clarity with which a risk perception is held, is also an emerging concept which has been proposed as a moderator of the relationship between risk perception and subsequent behaviour change [16]. For example, conceivably individuals who have a perception of high disease risk but who feel uncertain about this judgement, may be less motivated to engage in health promoting behaviours.

A further factor that may influence risk perception is the format in which risk is presented. There are over 2000 ways to present risk information [17]. While some studies have found no clear preference amongst participants on which should be used [18–21], some formats have been reported to have greater impact on accuracy or level of risk perception than others. For example, numerical presentation of risk as opposed to simple risk categories appears to lead to more accurate risk perception [22]; displaying risk information visually can enhance understanding compared with written information alone, particularly amongst those with low numeracy [23]; and pictographs are understood and interpreted with the greatest accuracy due to their clear display of the reference population to which the individual belongs [24, 25]. Presenting risk as a relative risk reduction also appears to be the most effective format for encouraging uptake of treatment [22], although this may be a function of the relative risk being confused for absolute risk. Presenting information about cancer risk in different formats may therefore result in different effects both on risk perception and behaviour.

In this parallel group, randomised controlled trial we aim to assess the short-term effect of providing information about personalised risk of cancer in three different formats alongside lifestyle advice on health-related behaviours, risk perception and risk conviction.

### Objectives

#### Primary objective

The primary objective is to evaluate the effect of providing different formats of personalised cancer risk information based on self-reported modifiable behaviour risk factors alongside lifestyle information on change from baseline in computed risk relative to an individual with a recommended lifestyle at three months.

#### Secondary objectives

The secondary objectives are to evaluate the effect of providing different formats of personalised cancer risk information alongside lifestyle information on: perceived risk of cancer; anxiety; cancer-related worry; intention to change behaviour; and awareness of cancer risk factors.

## Methods

### Study design

**Fig. 1:**
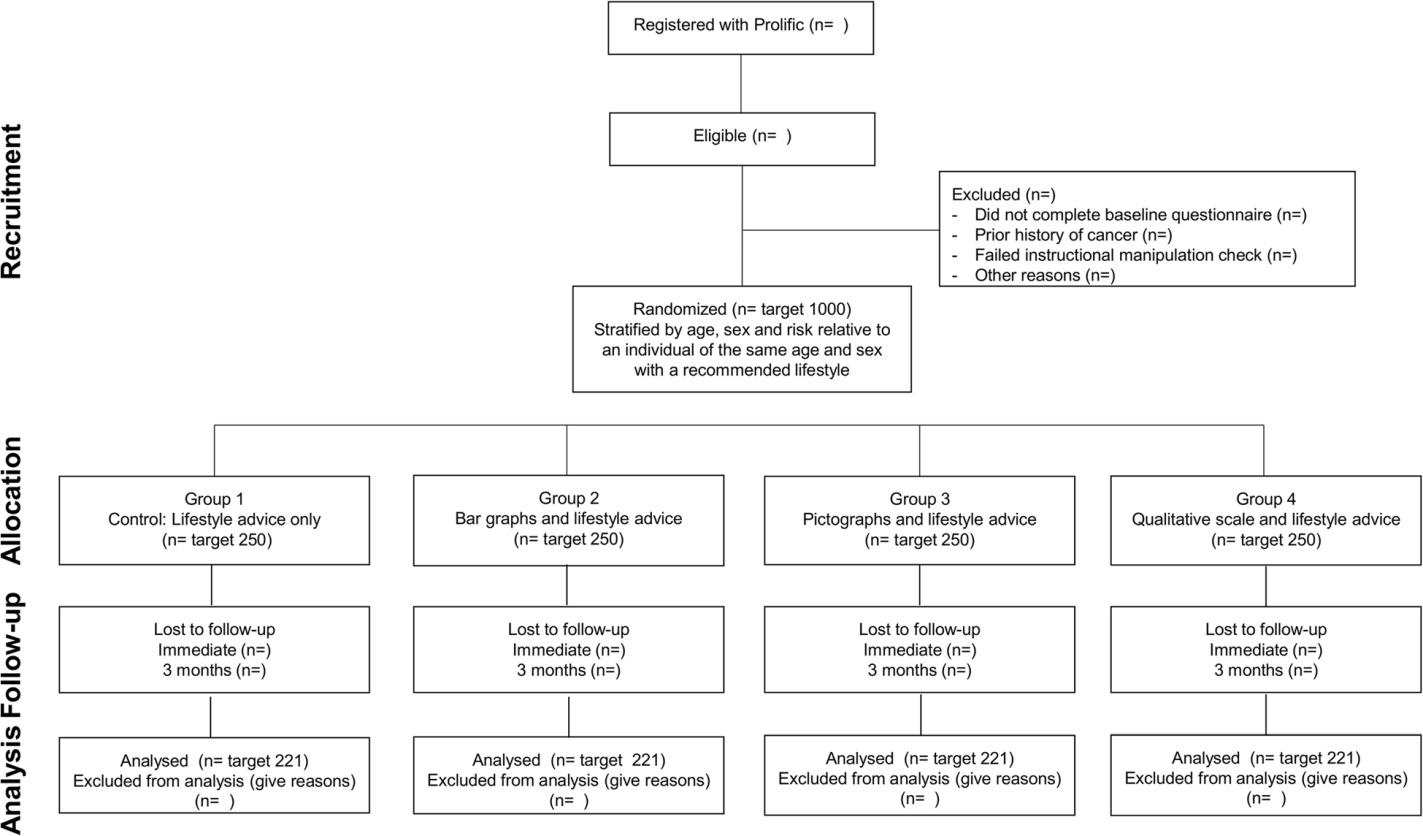
CONSORT diagram

The trial is a parallel group, randomised controlled trial with participants allocated to either a control group which receives cancer-specific lifestyle advice alone or one of three intervention groups which receive the same lifestyle advice alongside their estimated 10-year risk of developing one of the five most common preventable cancers in one of three different formats (bar chart, pictograph, qualitative scale). The design of the trial and flow of participants are shown in Fig. 1.

### Population and recruitment

We aim to recruit 1000 men and women between 30 and 74 years of age resident in the UK without a past history of cancer and with an approval rating ≥ 95% [26] using the Prolific platform (https://www.prolific.ac/). Prolific is an online participant recruitment platform for researchers in which participants volunteer to take part in studies and are compensated for their time with an agreed hourly rate. On 22 February 2018 there were 6245 participants registered with Prolific who met our inclusion criteria.

Eligible participants will be emailed an invitation to take part in the study by Prolific. If they are interested in taking part they will then be directed to the participant information sheet (Additional file 1) and have the opportunity to take part in the study or contact the research team for further information. They will be paid £2 for completing the baseline assessment and intervention and £1 for the follow-up 3 months later (the equivalent of £6/h). This will be paid through Prolific.

### Setting

The trial is online and led from the University of Cambridge. Participants are resident within the UK.

### Consent

Written online consent will be obtained from each participant at the start of the study and again at the beginning of the three month follow-up data collection.

### Baseline assessment

All baseline information will be collected prior to randomisation. After completing the electronic consent, participants will be directed to a baseline online questionnaire. This includes questions about age, sex, and current lifestyle (see below for details) to allow calculation of the individual’s cancer risk. The questions required for that calculation are compulsory. All other questions are optional. An instructional manipulation check is also included in the baseline questionnaire to identify inattentive participants and increase the validity and reliability of the responses [27–29]. This takes the form of a single question “*It is important that you pay attention in this study. Please tick ‘Strongly Disagree’.”* Participants who fail to answer the question correctly will be excluded from the study prior to randomisation.

### Randomisation, allocation concealment and blinding

The baseline questionnaires and all study materials will be developed within the Gorilla.sc research platform (www.gorilla.sc/about). Gorilla is a bespoke software program that allows researchers to develop online questionnaires and interactive tasks. Participants will be randomised 1:1:1:1 to the four groups at an individual level using a computer program built into the Gorilla research platform based on computer generated random numbers within block sizes of eight. Randomisation will be stratified by sex, risk relative to an individual with a recommended lifestyle (≤ or > 1.5) and age (≤ or > 40 years). Given the nature of the trial, it is not possible to blind participants to which intervention they receive. The researchers assessing the trial outcomes will remain blinded to the allocation of individuals until generation of the final dataset for analysis.

### Interventions

Participants in all four groups will be provided with tailored lifestyle advice. The three intervention groups will additionally be provided with a personalised estimate of their risk of developing cancer in one of three formats and have the opportunity to see the impact on their estimated risk of changes that they could make to their lifestyle. The control group will receive generic information about the link between lifestyle and cancer.

#### Lifestyle advice

All participants will be provided with links to web-based information on how to reduce risk of cancer through changes in lifestyle. This will include generic information about setting goals and obtaining support alongside specific information on each of the key target behaviours (quitting smoking, losing weight, reducing alcohol consumption, eating more fruit and vegetables, eating less red and processed meat and being more active). The links to these pages will be tailored so that individuals only see information relevant to them, for example non-smokers will not be provided with a link to information on smoking cessation. Each page relating to the target behaviours will include details of the association with cancer, the recommended daily or weekly amount, ideas about how to make changes, and a space for goal setting and action planning (see Additional file 2 for an example). Participants will be able to print each page separately for future reference. Clicking on all the links to the lifestyle information is not a requirement for completion of the trial. However, if a participant attempts to leave the page without viewing any lifestyle information a pop-up will appear saying “You have not viewed any of the lifestyle pages. You will not be able to return to this page. Are you sure you want to leave?”. A similar pop-up will appear reminding all participants that they will not be able to return to the lifestyle pages once they leave.

#### Risk estimates

The 10-year risk of developing one or more of the top five preventable cancers (lung, colorectal, bladder, kidney and oesophageal cancer for men and breast, lung, colorectal, endometrial and kidney cancer for women) will be estimated for each participant using a lifestyle-based risk score developed for this purpose. Full details of the development and validation of the model will be published separately. In brief, lifestyle factors for each cancer were selected from the European Code against Cancer 4th Edition [30–34] and estimates of relative risks obtained from meta-analyses of observational studies. Average population values of each risk factor in ten year age groups were obtained from nationally representative samples [35, 36] and mean 10-year estimated absolute risks from routinely available sources [37, 38]. Together these allowed us to calculate for each individual the absolute 10-year estimated risk and the 10-year risk relative to an average person of the same age and sex. To enable us to present estimates of the change in risk if individuals followed a “recommended” lifestyle, we also calculated the risk for each sex and ten year age group with recommended values of the risk factors. For smoking, body mass index (BMI, calculated as weight (kg) divided by the square of the height (m)), fruit and vegetable consumption and physical activity, we used the UK Department of Health guidelines to define these [39, 40] (being a non-smoker, having a BMI of 25 kg/m^2^, eating five portions of fruit and vegetables a day, and doing 150 min of moderate physical activity per week). For alcohol and red and processed meat consumption which are associated with increased risk, we used zero as our recommended level in line with recommendations from the World Cancer Research Fund [41]. This decision was made to avoid appearing to encourage consumption of red or processed meat or alcohol among those consuming small amounts.

The risk score included age, sex, BMI, fruit, vegetable, red meat and processed meat consumption, alcohol intake and physical activity. These were all obtained from participants’ responses in the baseline questionnaire.

#### Formats of risk presentation

**Fig. 2:**
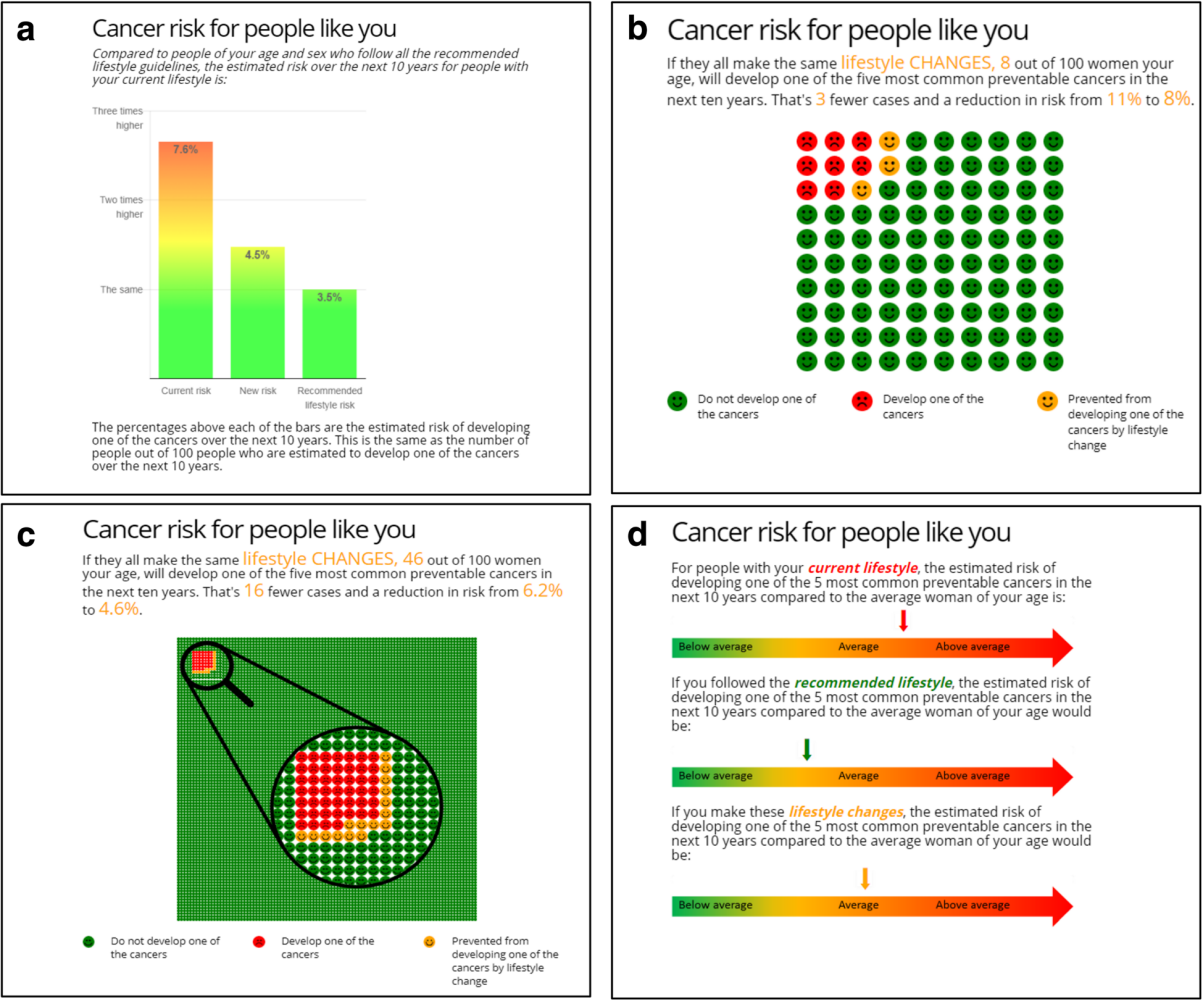
Risk presentation formats. (a) Bar chart; (b) 100 icon pictograph for those with an absolute risk > 8%; (c) 1000 icon pictograph with a magnified section of 100 icons for those with a relative risk ≤8%; (d) qualitative scale

In order to assess the effect of absolute and/or relative risk and numerical and/or verbal descriptions of risk, the three intervention groups will see their personalised risk estimate in one of three formats (Fig. 2). The first is a bar chart showing the risk for people with their current lifestyle compared to the risk for people of their age and sex who follow the recommended lifestyle, with estimates of absolute risk provided as percentages above each of the bars. The second is pictographs first showing the estimated absolute risk of people with their current lifestyle and then the difference between that and the absolute risk of people of their age and sex who follow a recommended lifestyle. To enable visualisation of the difference for those participants with a low absolute risk, those with an absolute risk > 8% are shown a 100 icon pictograph and those with a relative risk ≤8% a 1000 icon pictograph with a magnified section of 100 icons. The third provides qualitative information on a scale from “*Below average*” to “*Above average*” on the risk first for people with their current lifestyle compared to an average person of the same age and sex and then the risk for people of their age and sex who follow a recommended lifestyle compared to an average person of the same age and sex.

All three groups are then able to set target values for each of the lifestyle elements and see the impact of this on the risk estimates. They are unable to proceed to the next part of the trial without completing this step but can click back and forth to amend their target values and observe the effect on their risk estimate as many times as they wish. They can then print a summary of the risk estimates with their target values included.

### Post-intervention assessment

Immediately after viewing the lifestyle information or lifestyle information and risk presentation participants will be directed to complete a second online questionnaire. They will have a maximum of 45 min from providing initial consent to complete this. Approximately three months post-intervention, participants will be contacted through Prolific and invited to complete a final online questionnaire. On completion of that questionnaire those participants who were in the lifestyle advice only group will be given the opportunity to view their estimated risk and see in the impact of changes in lifestyle as for group two (bar chart presentation).

### Measures

The measures collected and the stage of the trial at which each is assessed are shown in Table 1. The primary outcome is change from baseline to three months in risk relative to an individual with a recommended lifestyle calculated from self-report data using the risk score described above. Participants have the option to enter height and weight in metric (m and kg) or imperial (feet and inches and stone and pounds) measures. Fruit and vegetable consumption are estimated from two questions: “How many portions of fruit/vegetables do you eat on a typical day?”. Each question is accompanied by images and descriptions of the rough equivalent of one portion for a range of fruit or vegetables. Red meat and processed meat consumption are collected using similar questions covering a typical week, again with images of a portion for a range of examples. Alcohol intake is assessed using the question “How many units of alcohol do you drink in a typical week?” with the number of units in a typical pint of beer or cider, a bottle of beer, a small or large glass of wine and a shot of spirits provided. Physical activity is estimated from responses to the question “How many hours of physical activity such as brisk walking, cycling, keep fit, aerobics, swimming or jogging, do you do in a typical week?”. Current and ex-smokers were identified from responses to the question “Do you currently smoke?” (*Yes, No, No but I used to*). Participants are not able to leave these questions blank. To improve the accuracy of responses to these questions a warning box appears alerting participants if their BMI is outside the range 12 to 50 kgm^− 2^ or if they have entered free text into any of the questions requiring a number. Participants then have the opportunity to correct their responses at that stage. This same check applies when data on these variables are collected at the three-month follow-up.

**Table 1:** List of outcome measures at each time point in the trial

Measure	Baseline	Immediately post intervention	3 months post intervention
Demographics			
Age	✓	–	–
Sex	✓	–	–
Ethnicity	✓	–	–
Family history of cancer	✓	–	–
Highest education level	✓	–	–
Lifestyle			
Self-reported weight	✓	–	✓
Self-reported height	✓	–	–
Smoking status (current/ex-smoking/never smoker)	✓	–	✓
Alcohol consumption (units per week)	✓	–	✓
Physical activity (hours per week)	✓	–	✓
Fruit consumption (portions per day)	✓	–	✓
Vegetable consumption (portions per day)	✓	–	✓
Red meat consumption (portions per week)	✓	–	✓
Processed meat consumption (portions per week)	✓	–	✓
Secondary outcome measures			
Awareness of cancer risk factors	✓	✓	✓
Risk perception	✓	✓	✓
Risk conviction	✓	✓	✓
Self-efficacy	–	✓	–
Response-efficacy	–	✓	–
Maladaptive coping	✓	✓	✓
Intention to change behaviour	–	✓	–
Worry (Lerman cancer worry scale)	✓	–	✓
Anxiety (short-item SSAI)	✓	✓	✓
Potential mediators and moderators			
Numeracy	✓	–	–
Time orientation	✓	–	–
Self-rated general health	✓	–	–
Previous information on risk of developing cancer	✓	–	–
Cognitive evaluation of provision of cancer risk scores:			
Acceptability/usefulness etc. of information	–	✓	–

All secondary outcomes and potential moderators and mediators are also measured via self-report. Perceived risk is measured using the tripartite risk perception (TRIRISK) model [15] which includes deliberative, affective and experiential components of perceived risk. After questions about both absolute and comparative risk, we will also collect data on risk conviction using two questions (‘How certain are you about your answer to the above question / How confident are you that the estimate you gave in response to [the question above] is accurate, that is, that it reflects your actual risk?’) on 7-point Likert response scales from 1 (*Not at all certain / Not at all confident) to 7 (Extremely certain / Extremely confident*). These questions are included to allow assessment of whether risk conviction improves how well the construct of perceived risk predicts behaviour. They are adapted from suggestions in the literature [16] and are currently also being tested in a study in the US (personal report).

We will also collect data on cancer-related worry and anxiety using the Lerman cancer worry scale [42, 43] and the short-form of the state scale of the Spielberger State Trait Anxiety Inventory (STAI) [44] respectively. The Lerman cancer worry scale has been widely used in the literature [45, 46] and the short-form STAI consists of 6 items that comprise the most highly correlated state anxiety-present and state anxiety-absent items from the full-form of the STAI. Scores obtained using this short-form have been shown to be highly correlated with scores obtained using the full-form of the STAI [44]. Maladapative behaviours will be assessed using three statements adapted from Rippetoe and Rogers [47] and awareness of cancer risk factors using question six from the Cancer Awareness Measure [48].

Overall intention to change behaviour will be measured immediately post intervention using four questions on 7-point Likert response scales from 1 (*Strongly disagree*) to 7 (*Strongly agree*) as in Ferrer et al.*,* [15] (‘I am determined to do everything I can to avoid getting cancer in the future.’/ ‘I am committed to engaging in behaviours that protect me against getting cancer in the future.’ / ‘I fully intend to have a lifestyle that will prevent me from getting cancer in the future.’ / ‘I will try to do all I can to avoid getting cancer in the future.’). Intention to change for each of the seven key lifestyle behaviours will also be asked separately using one item for each (e.g. ‘I intend to be more physically active in the next three months’) on a 5-point Likert response scale from 1 (*Strongly disagree*) to 5 (*Strongly agree*) with a sixth option ‘*Not applicable*’. Response efficacy and self-efficacy will both be measured using three items for physical activity and three items for diet as used in previous research [49, 50].

Self-rated health, family history of cancer, numeracy and time orientation will also be measured at baseline. Numeracy will be assessed using the 3-item Schwartz scale [51] adapted for the UK. Time orientation, the extent to which individuals tend to be motivated more by future or present goals when making decisions, will be measured using the brief nine item form of the Zimbardo Time Perspective Inventory (ZTPI-R) [52] which includes measures of both present and future orientation. We will also collect data on ethnicity and educational level in order to describe the cohort in comparison to the UK population.

Participants’ views of the risk and/or lifestyle information will also be assessed immediately post intervention by asking participants how much they agreed that the interventions were understandable, trustworthy, useful, motivating, important and well-presented and helped them decide about cancer risk reduction [53]. We will also collect process measures to assess how participants used the intervention using website analytics. These will include the total time spent on the intervention, the time spent viewing the risk information, the number of times participants set target values and view the effect of those changes on the estimates of risk, which lifestyle pages are viewed, and whether participants enter any specific behavioural goals.

### Statistical analyses

Univariate descriptive statistics (means and standard deviations or medians and interquartile ranges, numbers, and percentages) will be used to summarise participant characteristics at baseline overall and by randomised group and check for skewed distributions. All trial analyses will be performed including participants in the groups to which they were randomised (based on the intention-to-treat principle), but excluding individuals with missing outcome data.

For the primary outcome, intervention effects will be estimated using a linear regression model of change in risk relative to an individual with a recommended lifestyle (risk at three months follow-up minus risk at baseline), with the baseline value included as a covariate in the model (i.e. analysis of covariance, ANCOVA). The missing indicator method [54] will be used to enable individuals with missing values of the outcome at baseline to be included. An F-test will be performed of the null hypothesis that there is no difference between the four randomised groups. The model will also be used to derive estimates and confidence intervals from four pairwise comparisons: 1) Control group vs the three risk groups combined; 2) bar chart risk presentation vs pictographs; 3) bar chart risk presentation vs qualitative scale; and 4) pictographs vs qualitative scale. Multiplicative interactions between the interventions and each of the following variables will be tested using an F-test: age (≤ or > 40 years), sex, baseline risk relative to an individual with a recommended lifestyle (≤ or > 1.5), self-perceived risk at baseline below or above estimated risk or numeracy (< or ≥ two correct answers). If the *p*-value for interaction with one of the above variables is < 0.05, then estimates and confidence intervals for each of the pairwise comparisons will be derived within each subgroup defined by that variable.

Similar analyses will be used to estimate intervention effects on continuous secondary outcome variables. Smoking status (current vs ex-smoker and non-smoker) and accuracy (correct/incorrect) at three months follow-up will be analysed using binary logistic regression models, with adjustment for their respective values at baseline; randomised groups will be compared using a likelihood ratio test, and the four pairwise differences (risk ratios) and confidence intervals will be estimated. Intention to change behaviour, self-efficacy and response efficacy are only measured immediately after the intervention so linear regression will be used.

For all outcomes, 98.75% confidence intervals will be presented (based on a Bonferroni corrected significance threshold of 1.25%) to acknowledge the fact that four pairwise comparisons are presented. For any particular outcome, this is a conservative approach since the comparisons are not all independent of each other.

Acceptability, usefulness of the risk and/or lifestyle information, and process measures relating to use of the intervention will be summarised across the four groups using descriptive statistics (means, standard deviations, numbers, and percentages).

### Sample size

The target sample size is 1000 participants (250 per group). In the EPIC-Norfolk cohort [54] the mean risk of developing one or more of the five chosen cancers relative to an individual with a recommended lifestyle is 1.77 (SD 0.97). It is likely that the baseline and follow-up values of the outcome will be correlated, but the size of this correlation is unknown. To detect a baseline-adjusted between-group difference of 0.3 with a significance level of 1.25%, assuming the SD is 0.97 and 10% loss to follow-up, the power will be 79, 80% or 83% if the correlation is assumed to be 0.1, 0.2 or 0.3.

### Patient and public involvement (PPI)

Two patient and public representatives have been involved in the design of this trial. In particular, they have commented on the questionnaires and the wording and format of the presentation of the risk estimates, critically revised participant information sheets, and contributed to the development of the web-based lifestyle intervention. We expect that they will also be involved with developing newsletters and taking part in dissemination activities.

### Data management

Each participant is assigned a unique numeric identifier by Prolific so that no personal information is released to researchers. No personally identifiable data will be collected during the trial. All data will be held in accordance with the University of Cambridge Primary Care Unit policy on data security and confidentiality regulations will be strictly adhered to.

### Ethics

This trial is sponsored by the School of Clinical Medicine at the University of Cambridge. Ethical approval was received from the Psychology Research Ethics committee of the University of Cambridge on 12 December 2017 (Ref: PRE.2017.093). The trial was prospectively registered at the ISRCTN Registry (ISRCTN17450583) on 30 January 2018.

### Data monitoring

A data monitoring committee is not considered appropriate for this trial given the low-risk nature and the short period of time between recruitment and follow-up. The data are also both collected and stored online, so there are no risks in relation to data entry. The trial management committee, comprising the research team, will monitor the progress of the trial and ensure it runs in accordance with the protocol, oversee day-to-day management of the study and compliance with the Department of Health Research Governance Framework and the Guidelines for Good Clinical Practice. It will be also be responsible for communicating important protocol modifications to the sponsor, research ethics committee and ISRCTN registry.

### Dissemination

This trial is embedded within a larger programme of research developing and evaluating Interventions for Cancer Prevention in Primary Care (the I-CaPP programme).We plan to submit the findings of this trial to an open-access peer-reviewed journal and present the findings at national and international conferences. We will also send a summary of the findings to all participants and provide a summary on the I-CaPP website (http://www.phpc.cam.ac.uk/pcu/i-capp/).

## Discussion

This trial will provide much needed evidence of the short-term effects of communicating information about personalised risk of the five most common preventable cancers on risk-reducing health behaviours. It will also, to our knowledge, be the first to use the Tripartite model of risk perception in a UK population and the first to measure risk conviction both before and after provision of risk information.

The use of an integrated web-based platform to both deliver the intervention and collect data will also allow us to track the time spent on each page, the target values set, and individual participants’ routes through the intervention. While we do not plan a per protocol analysis because it is not possible for participants to proceed without viewing the risk information and setting targets, this will allow us to measure engagement with the intervention in detail [55] and explore which elements were used most by participants.

However, the chosen recruitment method, although enabling rapid recruitment of a large sample size, will inevitably limit generalisability due to the specific demographics of Prolific members. 72% of Prolific members are aged 20–40 years, 79% are Caucasian and 63% have A level or degree qualifications [56]. In addition members are seasoned research participants with experience of completing a variety of online tasks, and as such their computer literacy and aptitude for completing such tasks is likely to be above average compared to the general population [57].

Nevertheless, the findings of this trial have the potential to inform future work on development of risk-based interventions for cancer and guide the use of cancer risk scores online and within primary care.

## Additional files

Additional file 1 Participant information sheet and model consent form. (PDF 230 kb)

Additional file 2 Example of lifestyle information. (PDF 425 kb)

## Abbreviation

BMI: Body mass index
