# Supplementary material for: A randomised controlled trial of the effect of providing online risk information and lifestyle advice for the most common preventable cancers: study protocol
Source: BMC Public Health. 2018 Jun 26;18:796. doi: 10.1186/s12889-018-5712-2 (PMC6019532; doi:10.1186/s12889-018-5712-2)
Supplement: Supplementary file 1 — Participant information sheet and model consent form. (PDF 230 kb) [file 12889_2018_5712_MOESM2_ESM.pdf]

Losing weight

Strong evidence suggests that **excessive body fat** is linked to increased risk of **11** types of cancer. This includes breast, bowel and kidney.
Extra fat in the body can have harmful effects by increasing levels of hormones and growth factors which are known to promote the growth of cancer cells.

Carrying weight around your **abdomen**, having an "apple" body shape, can also put you at risk of other diseases and an even greater risk of developing cancer.

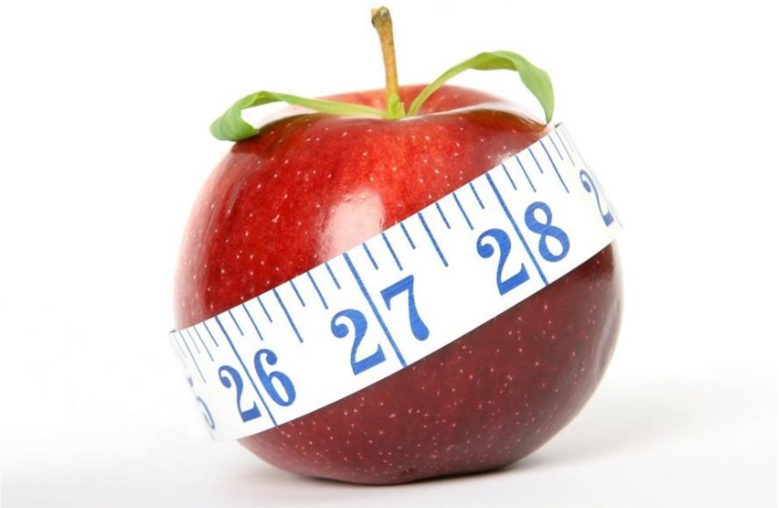

Therefore, achieving a healthy bodyweight can help to **reduce** your risk of cancer and other diseases.
Making small changes to your lifestyle that you are able to maintain can lead to weight loss.

It can be difficult to lose weight and keep it off but even small changes in your weight can have an impact on your overall health.

What changes could you make to reach a healthy bodyweight?

- Try to eat at the same times each day, to keep to a routine
- Have a glass of water before mealtimes
- Introduce fruit as a healthy snack to have between meals instead of crisps or chocolate
- Consider drinking water or sugar-free drinks
- Limit sugar added to cereal, tea and coffee
- Look at the fat and sugar content on food labels. The traffic light colour system will help you to know which foods are within a healthy range
- Be more active by walking, break up long periods of sitting

Use this space to write down your goal and plan how and when you will do your new action

....

....

....
